# Diagnosis-specific disability pension and risk of all-cause and cause-specific mortality – a cohort study of 4.9 million inhabitants in Sweden

**DOI:** 10.1186/1471-2458-14-1247

**Published:** 2014-12-04

**Authors:** Charlotte Björkenstam, Kristina Alexanderson, Emma Björkenstam, Christina Lindholm, Ellenor Mittendorfer-Rutz

**Affiliations:** Division of Insurance Medicine, Department of Clinical Neuroscience, Karolinska Institutet, SE 171 77 Stockholm, Sweden

**Keywords:** Mortality, Disability pension, Suicide, Cancer, Circulatory disease, Sick leave

## Abstract

**Background:**

The incidence of disability pension (DP) is high in several European countries. However, knowledge on associations of cause-specific DP and premature death is limited. The aims were to: 1) investigate the association between cause-specific DP and all-cause and cause-specific mortality among women and men and 2) examine period effects of this association.

**Methods:**

Three prospective population-based cohort studies were conducted, the first including all individuals aged 16–64 years who lived in Sweden all of 1995 and who were not on DP before 1995 (N = 5 006 523, 48.8% women). Those granted DP in 1995 were compared to those not granted DP regarding mortality during 1996–2009. Two other cohorts were created in a similar fashion, for 2000 and 2005, respectively, and in comparisons each of the three cohorts were followed up for four years with regard to all-cause mortality as well as death due to cancer, circulatory disorders, or suicide. All analyses were stratified by sex and we controlled for a number of socio-demographic factors and inpatient care.

**Results:**

Individuals with granted DP had a higher mortality risk, women (HR 1.75; 95% CI 1.68-1.82) and men (HR 1.66; 95% CI 1.61-1.71) and highest for cancer. People on DP with some diagnoses had higher risk of premature death in other causes of death than their DP diagnoses. All-cause mortality risk varied with DP-diagnosis and was lowest for musculoskeletal diagnoses. The mortality HR decreased among women with DP between the cohort 1995, HR 2.07 (1.92–2.24) and the cohort 2005, 1.84 (1.71–1.99). Here, temporal decreases in mortality risk occurred particularly in DP due to mental diagnoses and cancer.

**Conclusions:**

All DP diagnoses were associated with a higher mortality risk. Even individuals granted DP due to diagnoses with low mortality risk displayed a higher risk for premature death. This warrants close monitoring of disability pensioners and further studies on consequences of being on disability pension.

## Background

The incidence of disability pension (DP) continues to be high in many European countries, despite improvements in health and increases in life expectancy [1]. This development exacerbates not only the risk of shortage of labor and strains the economy of the society; for the individual, DP may also imply economic constraints, life style changes, social isolation, and development of other diseases, e.g., depression [2]. Although DP is common, there is very little knowledge on the life situation among people granted DP and especially so regarding specific DP diagnoses [2].

There is extensive research on possible consequences, in terms of ill-health and psychosocial aspects, of being long-term unemployed [3, 4]. Regarding DP, there are fewer studies and most of them focus on risk factors for DP, hardly any on the situation afterwards [2, 5]. This seems surprising, as physicians are involved in the process of granting DP, and healthcare usually is interested in possible consequences of a measure. A few studies have identified higher all-cause mortality among people on DP [6, 7]. The main dispute on this issue has focused on whether the higher mortality is inherently related to the underlying disease, or whether it is also associated with other factors, or even with the DP itself, that is, by being on DP, or its potential psycho-social consequences [7]. Some studies have shown that the underlying disease could only explain part of the higher mortality, other explanations are therefore warranted [7–9]. Potential mechanisms of an association between DP and subsequent mortality include being excluded from the labor market. Not being involved in gainful employment might be associated with a lack of daily routines and social contacts and even a lack of meaning [10]. Other potential mechanisms might include changes in health behavior, like increased alcohol and tobacco use and reduced physical exercise [11–13]. As it is not possible to study these associations by randomized controlled trials, other study designs are warranted, preferably large prospective cohort studies [5].

A Norwegian study of patients with musculoskeletal or mental diagnoses found DP to be a strong risk factor for premature death among both women and men [6]. Part of the higher risk was explained by DP due to disorders with a higher mortality rate, such as cancer or circulatory disease. However, in most cases DP is granted due to diseases that are not life threatening, e.g., musculoskeletal disorders. It is, therefore, urgent to further study the risk of premature death for specific DP diagnoses.

DP levels are affected by changes in economy, labor market situation, social insurance policies, unemployment rates, public health, healthcare policies, as well as employment frequencies among women and men and in different age groups [1, 14, 15]. Furthermore, particularly since the 1990s, several conditions that are potentially relevant for DP occurrences have changed [16, 17]. A recently published study found that young individuals who were granted DP between 2005 and 2006, had lower risk of suicide attempt before the DP compared with those granted during 1995–1997 and 2000–2002. Also the drop in risk of suicide attempt after the receipt of DP was less steep for the more recent cohort. One likely explanation of these findings is that individuals with less severe medical conditions are granted DP in more recent cohorts as compared with earlier ones [18]. Therefore, it is important to acknowledge temporal trends that affect rates and diagnostic profiles of DP and further elucidate the association between diagnosis-specific DP with cause-specific mortality. Few studies to date analyzed the association of DP with cause-specific mortality and most of them are restricted to study populations of specific occupational groups, ages, or regional areas [19, 20]. To the best of our knowledge, no study to date has investigated the association between diagnosis-specific DP and all-cause and cause-specific mortality covering a whole population and adjusting for various important confounders, including ill-health, while taking period effects into consideration. As both the incidence of DP and mortality differ between women and men we also considered eventual sex differences by stratifying the analyses by sex [5, 16, 21].

### Aim

The aims of this study were to 1) investigate the association between cause-specific DP and all-cause and cause-specific mortality, defined as death due to cancer, circulatory disorders, and suicide, among women and men, and 2) examine period effects of such associations, controlling for socio-demographic factors and morbidity by in-patient care.

## Methods

### Study population

Three population-based cohorts were followed prospectively. The first cohort included all individuals aged 16–64 years who were registered as living in Sweden 31 December 1994 and during the year 1995 and who were not on DP or old-age pension 1 January 1995 (N = 5 006 523, 48.8% women). All individuals granted DP sometime during 1995 were compared to individuals without DP in 1995 and the cohort was then followed from 1 January 1996 to 31 December 2009 (14 years), with regard to all-cause and cause-specific mortality. In order to analyze period effects, two additional cohorts were formed in the same manner, including all individuals aged 16–64 years and living in Sweden all of 2000 and 2005, respectively, and who were not on DP or old-age pension when the respective year begun. Individuals granted DP in 2000 and 2005, respectively, were compared to the individuals not on DP in 2000 or in 2005. The cohorts in 2000 and 2005 included 5 066 144 (48.7% women), and 5 072 599 individuals (48.2% women), respectively. In the analyses of period effects, all three cohorts were followed for four years, that is, the longest period the latest cohort could be followed.

The cohorts were defined using data from LISA, a longitudinal integrated population-based database for labor-market research, held by Statistics Sweden. By linking the unique personal identity number assigned to all Swedish residents [22], information was obtained on in-patient care and causes of deaths from the National Patient Register and the Cause of Death Register, both held by the National Board of Health and Welfare. Data on DP was obtained from the nationwide register MIDAS at the National Social Insurance Agency.

### Outcome – mortality

Outcome measures were all-cause mortality as well as death due to cancer, circulatory disorders, and suicide. Cause-specific mortality was classified according to the International Classification of Diseases (ICD) version 9 and 10 as follows: cancer (ICD-9: 140–239; ICD-10: C00-C97, D00-D48), circulatory disorders (ICD-9: 390–456; ICD-10: I00-I99) and suicide (ICD-9: E950-E959, E980-E989; ICD-10: X60-X84, Y10-Y34). When studying suicide, deaths with undetermined intent were included in order to reduce regional and temporal variation in ascertainment [23, 24]. Sensitivity analyses proved the comparability of the diagnoses.

### Exposure – DP

In Sweden, DP can be granted to all individuals who, due to disease or injury, have permanently reduced work capacity, even if not having had income from work. DP can be granted for full- or part-time absence and up until 65 years of age, the customary age of old-age retirement. Approximately 65% of the lost income, up to a limit, is covered by disability pension.

The exposure was defined as granted full- or part-time DP in the respective exposure years (1995, 2000, or 2005). The main DP diagnosis was used, classified according to ICD-9 and ICD-10, at chapter level for the eight main diagnostic groups: musculoskeletal disorders (ICD-9: 710–739; ICD-10: M00-M99), mental disorders (ICD-9: 290–319; ICD-10: F00-F99), respiratory disorders (ICD-9: 460–519; ICD-10: J00-J94), cancer (ICD-9:140–239; ICD-10: C00-C97, D00-D48), circulatory disorders (ICD-9: 390–456; ICD-10: I00-I99), endocrine disorders (ICD-9: 240–279; ICD-10: E00-E99), neurological disorders (ICD-9: 320–359; ICD-10: G00-G99), injuries and poisonings (ICD-9: E800-E99; ICD-10: V01-Y98), and other diagnoses (all other). Due to limited power, the analyses of period effects were restricted to the four most common diagnoses of DP: musculoskeletal, mental, circulatory, and cancer diagnoses.

### Covariates

Information on age, sex, family situation, socioeconomic position (measured by educational level), area of residence, and country of birth was also obtained from LISA. For categorization of the covariates, see Table 1. Previous health care consumption was measured as in-patient care (excluding maternity care without complications), categorized according to the median number of days of inpatient care during the five years preceding the study entry (1990–1994, 1995–1999, and 2000–2004, respectively) into three categories: no inpatient care, ≤ the median, and > the median. The median values for the three periods preceding the study entry of the cohorts were 4 days, 3 days, and 3 days, respectively.

**Table 1:** **Characteristics of the three studied cohorts including all individuals aged 16–64 years who lived in Sweden in 1995, 2000 and 2005, respectively, and who were not on disability pension (DP) at the beginning of the respective year (Cohort 1995, 2000, and 2005)**

	Cohort 1995				Cohort 2000				Cohort 2005			
	DP		no DP		DP		no DP		DP		no DP	
	n	%	n	%	n	%	n	%	n	%	n	%
**Sex**												
Women	20358	53.7	2421612	48.7	26208	57.9	2438910	48.6	35335	60.8	2410183	48.1
Men	17587	46.3	2546966	51.3	19094	42.1	2581932	51.4	22811	39.2	2604270	51.9
**Age group (years)**												
16-24	318	0.8	298600	6.0	580	1.3	295721	5.9	1273	2.2	340375	6.8
25-34	3017	8.0	1788560	36.0	3837	8.5	1682671	33.5	6148	10.6	1606313	32.0
35-44	5128	13.5	1118522	22.5	6952	15.3	1153019	23.0	10773	18.5	1182685	23.6
45-54	11125	29.3	1139937	22.9	12432	27.4	1102486	22.0	15444	26.6	1012138	20.2
55-64	18357	48.4	622959	12.5	21501	47.5	786945	15.7	24508	42.1	872942	17.4
**Family situation**												
Married/living with partner without children	12788	33.7	700484	14.1	14032	31.0	728505	14.5	15062	25.9	678992	13.5
Married/living with partner with children	8755	23.1	1939591	39.0	10423	23.0	1824464	36.3	15365	26.4	1809034	36.1
Single/divorced/separated/widowed without children	13482	35.5	1696103	34.1	16543	36.5	1817882	36.2	20610	35.4	1827298	36.4
Single/divorced/separated/widowed with children	2568	6.8	292175	5.9	3684	8.1	316839	6.3	5835	10.0	316827	6.3
Children living with parents, 16–20 years	352	0.9	340225	6.8	620	1.4	333142	6.6	1274	2.2	382279	7.6
Missing information	0	0.0	0	.0	0	00.0	10	0.0	0	0.0	23	0.0
**Educational level (years)**												
Elementary school (0–9)	16523	43.5	1257379	25.3	16483	36.4	1057075	21.1	15926	27.4	916305	18.3
Upper secondary school (10–12)	15899	41.9	2409220	48.5	21001	46.4	2444926	48.7	29392	50.5	2373077	47.3
Higher education (>12)	4901	12.9	1247676	25.1	7015	15.5	1471393	29.3	11909	20.5	1676687	33.4
Missing information	622	1.6	54303	1.1	803	1.8	47448	.9	919	1.6	48384	1.0
**Area of residence**												
Large cities	12920	34.0	1759306	35.4	14572	32.2	1860403	37.1	18481	31.8	1887748	37.6
Medium cities	12980	34.2	1759133	35.4	15692	34.6	1763245	35.1	20491	35.2	1775613	35.4
Small towns	12045	31.7	1450139	29.2	15038	33.2	1397194	27.8	19174	33.0	1351092	26.9
**Country of birth**												
Sweden	31083	81.9	4366386	87.9	36678	81.0	4374069	87.1	46949	80.7	4303132	85.8
Other Nordic countries	3069	8.1	187860	3.8	2954	6.5	163243	3.3	2897	5.0	142937	2.9
European countries, EU25, excluding Nordic countries	1443	3.8	103466	2.1	1431	3.2	101913	2.0	1513	2.6	104222	2.1
Rest of the world	2348	6.2	310539	6.3	4235	9.3	381310	7.6	6786	11.7	463847	9.3
Missing information	2	0.0	327	.0	4	0.0	307	.0	1	0.0	315	0.0
**Previous inpatient care, days**												
0 days	16443	43.3	3807908	76.6	20962	46.3	3933141	78.3	31244	53.7	4045285	80.7
≤ median^a^	5840	15.4	641118	12.9	6767	14.9	587024	11.7	9423	16.2	566677	11.3
> median^a^	15662	41.3	519552	10.5	17573	38.8	500677	10.0	17479	30.1	402491	8.0

^a^Median days in inpatient care during the five years preceding the study entry (1990–1994, 1995–1999, and 2000–2004, respectively) for cohort 1995 = 4, cohort 2000 = 3 and cohort 2005 = 3 days.

### Statistical analyses

Uni- and multivariate Hazard Ratios (HR) with 95% Confidence Intervals (CI) were derived from Cox proportional hazard regression models, analyzing the association between cause-specific DP and mortality. All analyses were stratified by sex due to the known sex differences in both DP and mortality [5, 16, 17, 25]. The reference group comprised women and men without DP during the respective exposure year. The individuals were followed up with regard to death, emigration, or to the end of the follow-up period, whichever came first. Missing information in any covariates was coded as a separate category. Statistical analyses were carried out using the SAS software package, version 9.2.

### Ethical considerations

This study was evaluated and approved by the Regional Ethical Review Board of Stockholm, Sweden.

## Results

Among individuals granted DP in 1995, there was a higher proportion of women (53.7%) than men (46.3%) and almost half were above 54 years old (48.4%) (Table 1). Twenty-five percent of individuals not granted DP in 1995 had only elementary school education compared to 43.5% among individuals granted DP. A majority of individuals with DP (57%) had at least one day of inpatient care 1990–1994 compared to 23% of those without DP. A higher number of both women and men were granted DP in 2005 than in 1995. The proportion of women among individuals granted DP increased during this period (53.7%, 57.9%, 60.8% in the cohort of 1995, 2000 and 2005, respectively). A minor shift in the age distribution from older to younger individuals granted DP from 1995 to 2005 could also be seen. Further, among disability pensioners the proportion with elementary school education decreased from 43.5% in 1995 to 27.4% in 2005, while the proportion with higher education increased from 12.9% to 20.5%. Changes in the distribution of educational level in the population without DP followed the same trends, but to a lesser degree.

The proportion of individuals on DP who were born outside Europe nearly doubled from 1995 to 2005 (6.2 versus 11.7, respectively). Also, the healthcare consumption varied across the three cohorts. While the proportion of disability pensioners without inpatient care increased from 1995 to 2005, the proportion of disability pensioners with an inpatient care stay exceeding the medium number of days in the preceding five years decreased. Similar changes in inpatient care use were seen for individuals without DP but not to the same extent.

In Table 2, the estimated HRs of mortality in the 1995 cohort for the 14 years of follow-up (1996–2009) related to DP diagnoses are shown. The incidence of DP was a little higher in women than in men. Musculoskeletal and mental diagnoses were the most common DP diagnoses among both sexes. The crude HR of all-cause mortality 1996–2009 was somewhat lower among women (HR 4.66; 95% CI 4.48-4.84) than among men (HR 5.86; 95% CI 5.68-6.04). Adjustment for age approximately halved the HRs among both sexes. After adjustments for in-patient care and socio-demographic factors there was still a higher mortality risk among individuals with DP compared to those without, but the sex difference disappeared; women (HR 1.75; 95% CI 1.68-1.82) and men (HR 1.66; 95% CI 1.61-1.71). Lack of sex differences in HRs for all-cause mortality was also observed for most of the DP diagnoses, with exception of DP due to respiratory diagnoses. While adjustment for socio-demographic factors changed the estimates related to diagnosis-specific DP only marginally, adjusting for in-patient care decreased the HRs considerably for all DP diagnoses. Risk estimates related to the different diagnostic DP groups in relation to mortality varied. Still, all DP diagnoses were associated with a higher mortality risk; lowest in DP due to musculoskeletal and highest in DP due to cancer.

**Table 2:** **Hazard ratios (HR) with 95% confidence intervals (CI) of all-cause mortality during 14 years of follow-up of all individuals aged 16–64 years who lived in Sweden in 1995 and were not on disability pension (DP) before that, by sex and DP with different diagnoses** ^**1**^

DP diagnoses	Population (n)	Deaths, n (%)	Hazard ratio (HR) with 95% CI, of death 1996-2009			
***Women***			Crude	Model I ^a^	Model II ^b^	Model III ^c^
No DP 1995	2421612	73138 (3.02)	1	1	1	1
DP granted 1995, all	20358	2723 (13.38)	4.66 (4.48–4.84)	2.22 (2.14–2.31)	2.07 (1.99–2.15)	1.75 (1.68–1.82)
Musculoskeletal	10181	898 (8.82)	2.97 (2.78–3.17)	1.30 (1.22–1.39)	1.21 (1.14–1.3)	1.09 (1.02–1.16)
Mental	3795	443 (11.67)	4.03 (3.67–4.42)	2.78 (2.54–3.06)	2.52 (2.30–2.77)	2.08 (1.90–2.29)
Circulatory	1200	251 (20.92)	7.57 (6.68–8.56)	2.79 (2.46–3.16)	2.68 (2.37–3.04)	1.89 (1.66–2.13)
Injuries and poisonings	751	73 (9.72)	3.31 (2.63–4.16)	1.85 (1.47–2.33)	1.77 (1.41–2.23)	1.34 (1.07–1.69)
Neurological	839	158 (18.83)	6.78 (5.8–7.93)	3.96 (3.39–4.63)	3.84 (3.28–4.49)	3.11 (2.66–3.64)
Respiratory	584	202 (34.59)	14.06 (12.24–16.14)	5.56 (4.84–6.38)	5.26 (4.58–6.04)	4.44 (3.86–5.09)
Cancer	587	296 (50.43)	27.25 (24.31–30.54)	11.41 (10.18–12.79)	11.08 (9.89–12.42)	7.36 (6.56–8.26)
Endocrine	457	130 (28.45)	10.96 (9.23–13.02)	5.20 (4.38–6.18)	4.70 (3.96–5.58)	3.66 (3.08–4.35)
Others	1964	272 (13.85)	4.84 (4.30–5.45)	2.23 (1.98–2.51)	2.09 (1.86–2.36)	1.75 (1.55–1.97)
***Men***						
No DP 1995	2546966	122720 (4.82)	1	1	1	1
DP granted 1995	17587	4 389 (24.96)	5.86 (5.68–6.04)	2.52 (2.44–2.59)	2.23 (2.16–2.30)	1.66 (1.61–1.71)
Musculoskeletal	6841	1188 (17.37)	3.83 (3.62–4.06)	1.46 (1.38–1.54)	1.30 (1.23–1.37)	1.11 (1.05–1.18)
Mental	3666	777 (21.19)	4.90 (4.57–5.26)	3.55 (3.31–3.81)	2.79 (2.59–2.99)	2.02 (1.89–2.17)
Circulatory	2620	966 (36.87)	9.28 (8.71–9.89)	3.13 (2.94–3.34)	2.91 (2.73–3.1)	1.72 (1.61–1.83)
Injuries and poisonings	865	155 (17.92)	4.01 (3.43–4.7)	2.03 (1.73–2.38)	1.83 (1.56–2.14)	1.18 (1.01–1.39)
Neurological	742	253 (34.1)	8.64 (7.63–9.77)	4.34 (3.84–4.91)	3.99 (3.52–4.51)	2.94 (2.60–3.33)
Respiratory	505	190 (37.62)	9.65 (8.37–11.13)	3.28 (2.84–3.78)	2.96 (2.57–3.42)	2.37 (2.05–2.73)
Cancer	375	250 (66.67)	27.38 (24.18–30.99)	11.44 (10.1–12.95)	10.96 (9.68–12.41)	6.39 (5.64–7.23)
Endocrine	502	254 (50.6)	14.54 (12.85–16.44)	5.76 (5.09–6.51)	5.16 (4.56–5.83)	3.40 (3.01–3.85)
Others	1471	356 (24.2)	5.69 (5.13–6.32)	2.45 (2.21–2.72)	2.25 (2.03–2.49)	1.72 (1.55–1.91)

^1^Analyses were stratified by sex; women and men without disability pension (DP) formed the reference groups, respectively.

^a^Model I: Adjusted for age.

^b^Model II: Adjusted for age, educational level, area of residence, country of birth, and family situation.

^c^Model III: Adjusted for age, educational level, area of residence, country of birth, family situation, and in-patient care.

In Table 3, HRs and CIs for cause-specific DP in relation to mortality due to cancer, circulatory disorders, and suicide are shown. In the multivariate analyses, mortality due to cancer was only associated with DP due to cancer or respiratory diagnoses. Death from circulatory disorders was associated with all DP diagnoses except cancer and injuries. Suicide was foremost associated with DP due to mental and musculoskeletal diagnoses in women and DP due to mental and neurological diagnoses among men.

**Table 3:** **Hazard ratios (HR) with 95% confidence intervals (CI) for mortality due to cancer, circulatory disorders, or suicide of all individuals (N = 5 006 523) aged 16–64 years who lived in Sweden in 1995 and not on disability pension (DP) before, during the follow-up 1996–2009, by DP diagnosis in 1995 and sex**

DP diagnoses	Death from cancer			Death from circulatory diseases			Death from suicide		
***Women***	n (%)	Crude HR	HR (95% CI) ^a^	n (%)	Crude HR	HR (95% CI) ^a^	n (%)	Crude HR	HR (95% CI) ^a^
No DP 1995	37973 (1.97)	1	1	11429 (0.47)	1	1	2159 (0.09)	1	1
DP all causes	1098 (5.39)	3.60 (3.39–3.82)	1.42 (1.33–1.51)	546 (2.68)	5.96 (5.47–6 49)	1.87 (1.71–2.04)	77 (0.38)	4.42 (3.52–5.55)	2.88 (2.28–3.63)
Musculoskeletal	410 (46.1)	2.60 (2.36–2.87)	0.99 (0.90–1.09)	185 (20.8)	3.90 (3.38–4.52)	1.18 (1.02–1.37)	19 (0.19)	2.12 (1.35–3.32)	1.58 (1.00–2.49)
Mental	112 (25.3)	1.95 (1.62–2.35)	1.08 (0.90–1.30)	86 (19.5)	4.99 (4.03–6.16)	2.36 (1.91–2.92)	46 (1.21)	14.05 (10.49–18.82)	7.23 (5.37–9.72)
Circulatory	69 (27.8)	3.96 (3.12–5.01)	1.06 (0.84–1.34)	104 (41.9)	19.90 (16.41–24.14)	3.96 (3.25–4.81)	< 7 (0.25)	3.01 (0.97–9.32)	1.62 (0.52–5.05)
Injuries and poisonings	30 (41.1)	2.61 (1.82–3.73)	1.12 (0.78–1.60)	14 (19.2)	4.05 (2.4–6.84)	1.40 (0.83–2.37)	< 7 (0.27)	3.05 (0.76–12.22)	1.85 (0.46–7.40)
Neurological	34 (21.7)	2.79 (1.99–3.90)	1.32 (0.94–1.85)	29 (18.5)	7.92 (5.5–11.41)	3.29 (2.29–4.75)	< 7 (0.12)	1.43 (0.20–10.18)	0.88 (0.12–6.28)
Respiratory	46 (22.9)	6.02 (4.51–8.04)	1.98 (1.48–2.64)	28 (13.9)	12.31 (8.49–17.83)	3.16 (2.18–4.58)	< 7 (0.34)	4.54 (1.14–18.18)	3.20 (0.8–12.83)
Cancer	268 (90.5)	46.47 (41.21–52.40)	13.54 (12.00–15.29)	7 (1.19)	4.08 (1.95–8.57)	0.91 (0.43–1.91)	0 (0)	0	0
Endocrine	22 (17.1)	3.51 (2.31–5.33)	1.27 (0.84–1.93)	45 (34.9)	24.04 (17.94–32.21)	6.48 (4.83–8.69)	< 7 (0.22)	2.78 (0.39–19.73)	1.58 (0.22–11.21)
Others	107 (40.2)	3.64 (3.01–4.40)	1.40 (1.15–1.69)	48 (18.0)	5.44 (4.1–7.23)	1.60 (1.20–2.12)	< 7 (0.15)	1.79 (0.58–5.56)	1.17 (0.38–3.65)
***Men***									
No DP 1995	39881 (1.57)	1	1	33081 (1.30)	1	1	6131 (0.24)	1	1
DP all causes	1158 (6.58)	4.72 (4.45–5.00)	1.41 (1.32–1.49)	1476 (8.39)	7.23 (6.86–7.62)	1.78 (1.69–1.88)	117 (0.67)	3.06 (2.55–3.68)	1.76 (1.46–2.13)
Musculoskeletal	402 (34.4)	3.97 (3.60–4.38)	1.13 (1.02–1.24)	373 (31.9)	4.43 (4–4.91)	1.11 (1.00–1.23)	21 (0.31)	1.34 (0.87–2.05)	0.90 (0.59–1.39)
Mental	119 (15.6)	2.30 (1.92–2.75)	1.19 (0.99–1.43)	187 (24.5)	4.34 (3.76–5.01)	1.67 (1.44–1.93)	69 (1.88)	8.57 (6.76–10.87)	4.18 (3.29–5.32)
Circulatory	189 (19.8)	5.51 (4.77–6.35)	1.10 (0.95–1.27)	524 (54.8)	18.33 (16.81–19.98)	2.87 (2.63–3.14)	7 (0.27)	1.30 (0.62–2.73)	0.64 (0.3–1.34)
Injuries and poisonings	37 (24.7)	2.93 (2.12–4.04)	0.96 (0.70–1.33)	41 (27.3)	3.91 (2.88–5.31)	1.01 (0.75–1.38)	7 (0.81)	3.58 (1.71–7.51)	1.81 (0.86–3.8)
Neurological	40 (15.9)	4.15 (3.04–5.66)	1.50 (1.10–2.05)	70 (27.8)	8.72 (6.9–11.03)	2.65 (2.09–3.35)	7 (0.94)	4.64 (2.21–9.73)	2.68 (1.27–5.62)
Respiratory	43 (22.9)	6.62 (4.91–8.93)	1.60 (1.18–2.15)	48 (25.5)	8.87 (6.68–11.77)	1.86 (1.40–2.47)	< 7 (0.2)	0.98 (0.14–6.96)	0.63 (0.09–4.48)
Cancer	205 (82.3)	67.57 (58.90–77.51)	17.05 (14.85–19.59)	18 (7.2)	7.08 (4.46–11.24)	1.43 (0.90–2.28)	< 7 (0.27)	2.07 (0.29–14.7)	1.07 (0.15–7.58)
Endocrine	36 (14.3)	6.21 (4.48–8.61)	1.58 (1.14–2.19)	112 (44.6)	23.14 (19.22–27.85)	4.65 (3.86–5.60)	< 7 (0.2)	1.09 (0.15–7.71)	0.56 (0.08–3.99)
Others	87 (25.7)	4.25 (3.44–5.24)	1.32 (1.07–1.63)	103 (30.5)	6.05 (4.98–7.34)	1.60 (1.32–1.94)	< 7 (0.2)	0.94 (0.3–2.92)	0.59 (0.19–1.82)

^a^Adjusted for age, education level, area of residence, country of birth, family situation, and in-patient care.

Tables 4 and 5 show HRs for women and men with DP due to different diagnoses for all-cause mortality for the three different cohorts (1995, 2000, 2005). The incidence of DP increased considerably between the cohorts 1995 and 2005, especially among women (Table 4 and Table 5). The mortality risk during the four-year follow up of each cohort was elevated in women granted DP compared to women not granted DP. The crude risk decreased between all the cohorts; HR 6.33 in 1995, 5.29 in 2000, and 4.58 in 2005. After adjustments, estimates decreased considerably but remained significantly associated with mortality. Further, the decrease of the estimates from 1995 to 2005 remained after multivariate adjustment.

**Table 4:** **Hazard ratios (HR) of all-cause mortality with 95% confidence intervals (CI) for women of the 1995, 2000, and 2005 cohorts, respectively, during 4 years follow-up, by disability pension (DP) diagnoses**

Women	Disability pension n	Death		Crude HR (95% CI)	Model I ^a^HR (95 CI)	Model II ^b^HR (95% CI)	Model III ^c^HR (95% CI)
		N	%				
*No DP*				1	1	1	1
*Granted DP 1995*	20 358	675	3.32	6.33 (5.86–6.84)	3.07 (2.84–3.32)	2.86 (2.64–3.09)	2.07 (1.92–2.24)
*2000*	26 208	732	2.79	5.29 (4.91–5.70)	2.79 (2.59–3.00)	2.60 (2.42–2.81)	1.91 (1.77–2.06)
*2005*	35 335	759	2.15	4.58 (4.26–4.93)	2.64 (2.45–2.84)	2.48 (2.31–2.67)	1.84 (1.71–1.99)
*Musculoskeletal*							
1995	10 181	132	1.30	2.44 (2.06–2.90)	1.10 (0.93–1.30)	1.02 (0.86–1.21)	0.84 (0.70–0.99)
2000	12 109	176	1.45	2.73 (2.36–3.17)	1.32 (1.13–1.53)	1.23 (1.06–1.42)	1.02 (0.87–1.18)
2005	13 178	153	1.16	2.46 (2.10–2.88)	1.26 (1.07–1.48)	1.16 (0.99–1.36)	0.96 (0.82–1.13)
*Mental*							
1995	3 795	103	2.71	5.16 (4.25–6.26)	3.57 (2.94–4.33)	3.19 (2.63–3.87)	2.23 (1.84–2.71)
2000	6 047	134	2.22	4.19 (3.53–4.96)	2.92 (2.46–3.46)	2.62 (2.21–3.11)	1.91 (1.61–2.27)
2005	12 887	155	1.20	2.55 (2.18–2.99)	1.84 (1.57–2.16)	1.77 (1.51–2.07)	1.41 (1.20–1.66)
*Circulatory*							
1995	1 200	47	3.92	7.48 (5.62–9.96)	2.83 (2.13–3.78)	2.70 (2.03–3.6)	1.47 (1.10–1.96)
2000	1 323	55	4.16	7.94 (6.09–10.34)	3.24 (2.48–4.22)	3.04 (2.34–3.97)	1.61 (1.23–2.10)
2005	1 321	54	4.09	8.78 (6.72–11.47)	3.56 (2.73–4.65)	3.32 (2.54–4.34)	1.66 (1.27–2.18)
*Cancer*							
1995	587	192	32.71	79.17 (68.66–91.30)	34.18 (29.62–39.43)	33.04 (28.64–38.13)	16.59 (14.35–19.17)
2000	668	186	27.84	63.47 (54.91–73.35)	28.72 (24.84–33.21)	27.77 (24.02–32.11)	13.80 (11.92–15.98)
2005	886	224	25.28	63.95 (56.02–72.99)	28.64 (25.09–32.71)	28.04 (24.56–32.02)	12.98 (11.34–14.86)

^a^Adjusted for age.

^b^Adjusted for age, education level, area of residence, country of birth, and family situation.

^c^Adjusted for age, education level, area of residence, country of birth, family situation, and in-patient care.

**Table 5:** **Hazard ratios (HR) of all-cause mortality with 95% confidence intervals (CI) for men from the cohorts of the 1995, 2000, and 2005, respectively, during 4 years follow-up, by disability pension (DP) diagnoses**

***Men***	Disability pension	Death in 4 year follow-up			Risk (HR) of death in four years follow-up		
	(n)	n	%	Crude HR (95% CI)	Model I ^a^HR (95% CI)	Model II ^b^HR (95% CI)	Model III ^c^HR (95% CI)
No DP in resp. cohort				1	1		
*Granted DP in 1995*	17 587	1 136	6.46	7.47 (7.03–7.93)	3.40 (3.20–3.61)	2.96 (2.79–3.15)	1.92 (1.81–2.04)
*Granted DP in 2000*	19 094	1 115	5.84	6.75 (6.36–7.17)	3.48 (3.28–3.70)	2.99 (2.81–3.17)	1.88 (1.77–2.00)
*Granted DP in 2005*	22 811	1 187	5.20	6.65 (6.27–7.05)	3.80 (3.58–4.03)	3.32 (3.13–3.52)	2.22 (2.09–2.36)
DP–diagnosis							
*Musculoskeletal*							
1995	6 841	224	3.27	3.71 (3.25–4.23)	1.51 (1.33–1.73)	1.33 (1.17–1.52)	1.06 (0.93–1.21)
2000	6 297	206	3.27	3.73 (3.25–4.27)	1.68 (1.46–1.93)	1.45 (1.26–1.66)	1.12 (0.98–1.28)
2005	6 988	207	2.96	3.73 (3.25–4.28)	1.82 (1.59–2.09)	1.59 (1.39–1.83)	1.27 (1.11–1.46)
*Mental s*							
1995	3 666	213	5.81	6.70 (5.85–7.67)	4.90 (4.28–5.61)	3.70 (3.23–4.24)	2.32 (2.02–2.66)
2000	4 952	279	5.63	6.52 (5.79–7.33)	5.18 (4.6–5.83)	4.02 (3.57–4.53)	2.56 (2.27–2.88)
2005	7 778	271	3.48	4.40 (3.91–4.96)	3.62 (3.21–4.08)	3.09 (2.74–3.49)	2.17 (1.92–2.44)
*Circulatory*							
1995	2 620	217	8.28	9.64 (8.44–11.02)	3.51 (3.07–4.02)	3.21 (2.81–3.67)	1.58 (1.38–1.81)
2000	2 441	187	7.66	8.92 (7.72–10.30)	3.50 (3.03–4.05)	3.10 (2.68–3.58)	1.44 (1.25–1.67)
2005	2 096	141	6.73	8.68(7.35–10.24)	3.52 (2.98–4.16)	3.10 (2.63–3.67)	1.50 (1.27–1.77)
*Cancer*							
1995	375	126	36.80	55.48 (46.93–65.58)	23.63 (19.98–27.94)	22.43 (18.97–26.53)	10.38 (8.77–12.29)
2000	460	130	28.26	39.22 (33.01–46.60)	17.73 (14.92–21.07)	15.80 (13.29–18.78)	7.14 (6.00–8.49)
2005	505	194	38.42	64.62 (56.10–74.43)	27.94 (24.24–32.19)	25.76 (22.35–29.68)	12.34 (10.70–14.24)

^a^Adjusted for age.

^b^Adjusted for age, education level, area of residence, country of birth, and family situation.

^c^Adjusted for age, education level, area of residence, country of birth, family situation, and in-patient care.

The incidence of DP due to musculoskeletal diagnoses increased slightly from the first cohort 1995 to the 2005 cohort among women (Table 4). After multivariate adjustment, DP due to musculoskeletal diagnoses was not associated with a higher mortality risk in any of the three cohorts among women. Table 4 also demonstrates a substantial increase in the number of women granted DP due to mental diagnoses between the cohorts, from 3 795 in the 1995 cohort to 6 047 in the middle cohort (2000), and 12 887 in the last cohort, 2005. Among individuals granted DP, the HRs decreased from HR 2.23 (95% CI 1.84-2.71) in the first cohort (1995) to HR 1.41 (95% CI 1.20-1.66) in the last one (2005), in the fully adjusted models.

Women granted DP due to cancer had a higher (crude) mortality risk in all three cohorts and after multivariate adjustment the estimates were still high: HR 16.59 (95% CI 14.35-19.17) in the 1995 cohort, 13.80 (95% CI 11.92–15.98) in the second cohort (2000), and 12.98 (95% CI 11.34–14.86) in the last cohort, 2005. The only DP diagnosis for which the estimates showed slight increases between the first and last cohort, both regarding DP incidence and mortality risk was DP due to circulatory diagnoses. Adjustment for in-patient care showed decreasing effects over time.

Among men, being granted DP was associated with a higher all-cause mortality risk in all three cohorts. The adjustments, particularly for age, had, however, a different impact on the estimates in men than in women. The fully adjusted HRs slightly increased between the cohort 1995; HR 1.92, (95% CI 1.81-2.04) and the last cohort of 2005; HR 2.22 (95% CI 2.09-2.36). As for women, the mortality risk varied with DP diagnosis; highest mortality risk was seen in DP due to cancer and lowest in DP due to musculoskeletal diseases.

An increase in the number of individuals granted DP between the cohorts was seen for mental diagnoses also among men, however, not to the same extent as for women: 3 666 in the 1995 cohort, 4 952 in the second cohort (2000), and 7 778 in the last cohort, 2005. The mortality risk estimates related to DP due to mental diagnoses were considerably affected by adjustment for socio-demographic factors particularly in the first (1995) and in the last (2000) cohort. Adjustment for in-patient care had an impact on the risk estimates in all three cohorts.

Among men with DP due to circulatory diagnoses, estimates showed a slight decrease between the cohorts both in DP incidence and in mortality risk. Adjustment for age and in-patient care showed also for these diagnoses a significant impact on the estimates. The number of men with DP due to cancer increased from 375 in 1995, 460 in 2000, to 505 in 2005. Among men, the mortality risk decreased between 1995 and 2000, multi-adjusted HR: 10.38 (95% CI 8.77–12.29) in 1995 and 7.14 (95% CI 6.00–8.49) in 2000, and then increased to HR 12.34 (95% CI 10.70–14.24) in 2005. Adjustment for age and in-patient care showed a similar impact on the estimates in all three cohorts.

## Discussion

The results from this prospective and population-based cohort study with a 14-years follow-up period including 4.9 million women and men from the general population of working ages, clearly demonstrate a higher mortality risk among people granted DP. Moreover, the risks were similar among women and men after adjustments for age, socio-economic factors, and in-patient care. The risks for premature death in the three studied specific causes of death varied with DP diagnosis. The period effect between the three cohorts of 1995, 2000, and 2005 showed a decreased risk of all-cause mortality between the cohorts in the respective four-years follow-up period among women, however, not among men.

We found a 75% and a 66% higher risk of all-cause mortality in women and men granted DP in 1995. This is in line with the findings from a Danish study including all individuals born between 1926 and 1936 showing a 2-fold (slightly higher in men than in women) higher mortality risk in people granted DP compared to the general population [26]. Previous research has shown the importance of age as a confounder for mortality among disability pensioners [8]. In a study of 245 704 individuals who had a new spell of long-term sickness absence lasting ≥56 days, 1985–1987 and followed to 1996, age-stratified analyses showed the mortality risk to be high among disability pensioners in general and among the younger ones in particular, compared with individuals without DP [7]. Our results, when adjusted for age halved the HR for all cause and cause-specific mortality among women and men with DP in general and also when analyzing DP diagnosis, however, with variation between the diagnoses.

Individuals on DP are a selected group of people having a disease or injury causing permanent work incapacity. The diagnoses and the severity thereof vary and make individuals on DP a heterogeneous group. The variation in mortality risk between different DP-diagnoses seen in our study is in line with two previous studies [9, 27]. The increased general mortality risk among individuals granted DP might not be surprising as they are in fact too ill to work. What is more interesting though is that individuals granted DP due to non-fatal diagnoses such as musculoskeletal diagnoses still showed higher mortality risks, also when controlling for inpatient care. Therefore, residual confounding through unmeasured morbidity is likely.

Which mechanisms that underlie the higher mortality is not yet fully explored, although some studies have attempted to clarify the higher risk [9, 26, 28, 29]. As a previous study on smoking and DP showed that smokers are at considerably higher risk of early retirement due to chronic disease [30], it is adequate to ask if smoking also could be an important factor in explaining the higher mortality risk in individuals granted DP, either through being a reason for the disease leading to DP, or through a change of life style during DP. A prior Swedish study found the mortality among DP pensioners to be especially high the first year of DP [8]. Another study on Norwegian data confirmed a high risk of premature death among disability pensioners in the period 1990–1996. The authors concluded that the medical condition seemed to contribute more to the higher mortality among women, whereas a low socio-economic status was more important for men [31]. More research is needed to investigate what mechanisms underlie these associations.

We found that the analyzed specific causes of death (cancer, circulatory disorder, and suicide) were not only higher among those with DP due to the related diagnoses, e.g. DP due to cancer and cancer death, but also in a number of other DP diagnoses. This was particularly the case for death due to circulatory disorder. In the analyses only the main DP diagnosis was used, and future studies are warranted to scrutinize possible associations with comorbidity in individuals with DP. The risk of suicide was higher in DP due to musculoskeletal, neurological, and particularly mental diagnoses, which is in line with previous findings [27]. Still we did not find higher risk for suicide among people with DP due to cancer, although cancer patients have been shown to have higher suicide risks [32].

During the time period 1995–2005 we found an overall decrease in risk of premature death in women and men granted DP. The incidence of DP increased in both women and men during the same time period, and more strongly in women. In addition, an almost 3-fold increase in DP among the youngest age group was found between the cohorts. The incidence of DP is among other factors affected by changes in unemployment rates and social insurance policies. After the 90’s recession the unemployment rate was high in Sweden in 1995 [33]. Between 1995 and 2005 the Swedish unemployment rate steadily decreased. In addition, since the 1990s and particularly in 2003 policies for being granted DP, especially when below the age of 30 years, have changed considerably in Sweden, leading to increasing rates [34]. The huge expansion of DP granted due to mental diagnoses between the cohorts combined with the related decreased mortality risk among women may indicate that the expansion was due to DP with less severe mental disorders. Our results did, however, not show a similar decrease in mortality risk in men granted DP due to mental diagnoses. The reason thereof is beyond the aims of this study, but needs to be further investigated.

Based on our results, DP could be seen as a risk indicator for premature death. There are several theories on how DP could affect mortality risks, besides the risk associated with the underlying disease e.g., related to changes of life style, worsening economy, social isolation, loss of the positive effects of paid work, or that other diseases develop due to these factors, e.g., depression [2]. However, there is no evidence regarding these mechanisms [2] and it is challenging to design a scientific study disentangling the consequences of the disease from the consequences of being on DP due to that disease. RCTs can hardly be conducted due to ethical reasons and other study designs will inherently be challenged by residual confounding and confounding by indication.

The strengths of this study include the prospective population-based cohort design and the large cohort including all individuals aged 16–64 years in Sweden at risk of DP. Hence, we had a good opportunity to study subgroups with significant estimates. Another strength is the long follow-up period that is required to obtain significant estimates even among those for whom premature death is rare. A further strength includes the analyses of mortality risks for three time periods with similar length of follow-up, which allowed estimation of consistency over time periods. Additional strengths are the high quality of the nationwide registers [35], not self-reports, and that there was no loss to follow up.

We adjusted for morbidity, through using data on previous in-patient care – this can be seen as both a strength and a limitation; a strength as only the more severe types of morbidity were adjusted for, and a limitation as different types of morbidity thus were not included. Therefore, residual confounding through unmeasured morbidity is likely. However, there is no nationwide out-patient register covering all those years. Also other limitations should be mentioned. Data on DP-diagnoses included only the main diagnosis of the granted DP. We were, therefore, not able to adjust for co-morbidity, which could overestimate the mortality risk for the specific DP diagnosis. Further, in this study we had no self-reported information on smoking or other health behaviors and could therefore not control for that. However, educational level is adjusted for and that often interacts with health behaviors. The associations between DP and premature death are probably underestimated as many people in the reference group were granted DP during follow up, some even as soon as the following year.

The main part of the higher mortality risk among people granted DP observed in this study was explained by age. However, people on DP still had 70% higher risk of premature death compared to people not granted DP the respective years, after further adjustments for other socio-demographics and morbidity measured as inpatient care. The confounding factors, especially previous in-patient care, seemed to explain more of men’s mortality risk than women’s. The confounding effect varied, however, between the time periods.

## Conclusions

In conclusion, this study clearly demonstrates a higher mortality risk among people granted DP. Even individuals granted DP due to diagnoses with low mortality risk displayed a higher risk for premature death – and that in spite of the fact that some individuals in the reference group could have been granted DP as early as the year following exposure. This warrants close monitoring of individuals on DP and further studies on consequences of DP.

## Funding

This study was financially supported by the Swedish Ministry of Health and Welfare, the Swedish Research Council for Health, Working life and Welfare, and the Swedish Research Council.
